# Correlation profiles between interoception and exteroception in psychotic patients versus healthy controls

**DOI:** 10.1192/j.eurpsy.2021.1430

**Published:** 2021-08-13

**Authors:** A. Donadeo, P. Politi, A. Silva, S.C. Civardi, E. Farinella, N. Brondino, M. Olivola, F. Sommi, S. Damiani

**Affiliations:** Department Of Brain And Behavioral Sciences, University of Pavia, Pavia, Italy

**Keywords:** psychosis, sensory profile, Interoceptive Awareness, psychopathology

## Abstract

**Introduction:**

Individual abilities to perceive internal and external sensations are defined respectively as interoception and exteroception: the dysregulation of these functions can explain many psychotic symptoms. (Ardizzi et al. 2016)

**Objectives:**

We evaluated the differences in the interoceptive and exteroceptive perception between 39 patients with psychosis and 250 healthy controls using self-administered questionnaires. The association between interoception and exteroception in the two groups was also tested.

**Methods:**

The tests we used are AASP (Adolescent / Adult Sensory Profile) and MAIA (Multidimensional Assessment of Interoceptive Awareness). Differences were measured with t-tests, associations with spearman’s correlation.

**Results:**

Significant differences emerged between the two samples in the AASP total score and in its Low registration (LR) and Sensory Avoiding (SA) sub-scales and in the MAIA total score and in all its sub-scales except “Not Worrying” (Fig.1). Different patterns of associations between AASP and MAIA were observed: psychotic patients showed negative correlations between MAIA and AASP in the LR and Sensation Seeking (SS) sub-scales and in the auditory (AU) and tactile (TO) sensory channels. Healthy controls, positive correlations emerged between MAIA and AASP in the Sensation Seeking (SK) sub-scale and in the “perception of movement” (MO) sub-score (Fig.2)(Fig.3).

**Figure:**
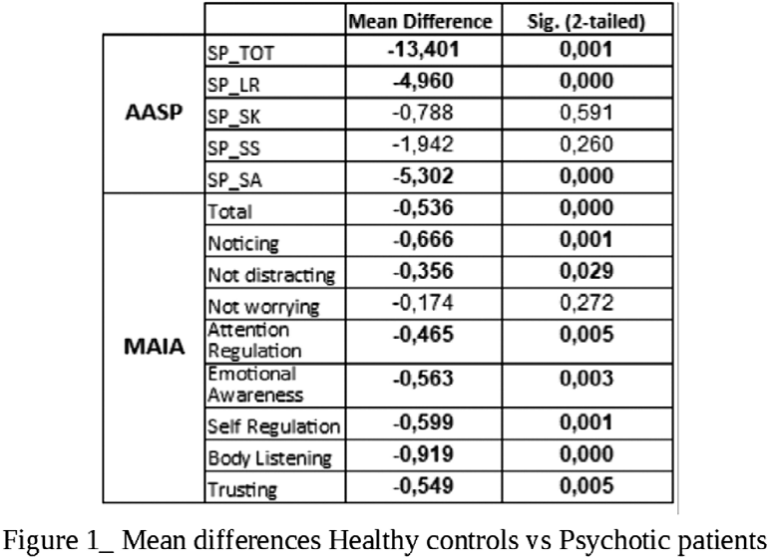

**Figure:**
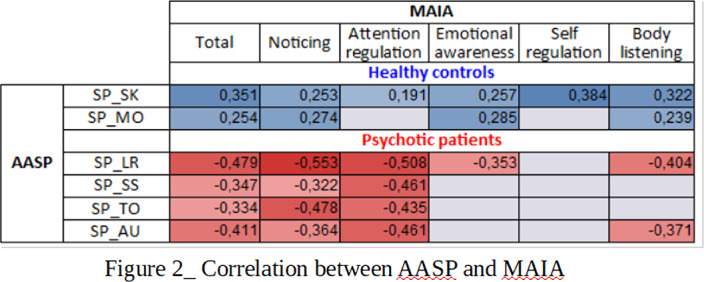

**Figure:**
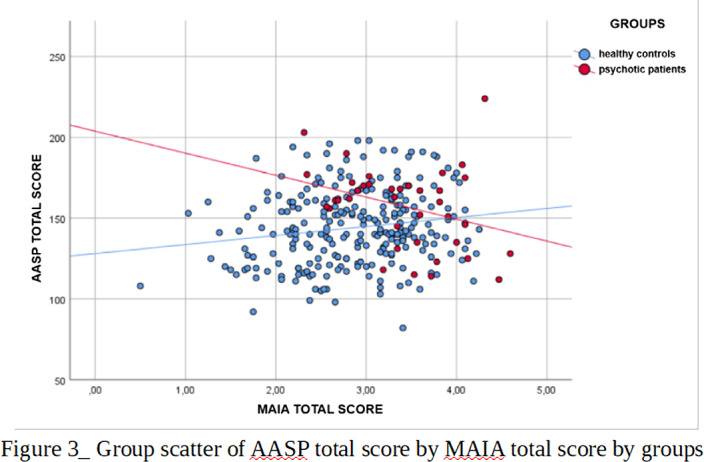

**Conclusions:**

Higher scores of psychotic patients in AASP and MAIA reveal both a disregulated sensory related behavior and a hightened awareness towards internal stimuli. The negative correlation between the two scales in psychotic subjects highlights the importance of the interaction between internal and external perception in determining the global subjective experience.

